# Metagenomic 16S rDNA amplicon data of microbial diversity of guts in Vietnamese humans with type 2 diabetes and nondiabetic adults

**DOI:** 10.1016/j.dib.2020.106690

**Published:** 2020-12-24

**Authors:** Hung The Hoang, Duc Hoang Le, Thi Thanh Huyen Le, Thi Tuyet Nhung Nguyen, Ha Hoang Chu, Nam Trung Nguyen

**Affiliations:** a National Key Laboratory of Gene Technology, Institute of Biotechnology, Vietnam Academy of Science and Technology, Cau Giay, Hanoi, Vietnam; b Graduate University of Science and Technology, Vietnam Academy of Science and Technology, Cau Giay, Hanoi, Vietnam; c Military Academy of Logistics, Long Bien, Hanoi, Vietnam; d Transport Hospital Joint Stock Company, Dong Da, Hanoi, Vietnam

**Keywords:** Human gut microbiota, Bacterial composition, Type 2 diabetes mellitus, Illumina sequencing

## Abstract

Type 2 diabetes mellitus (T2DM) is an important public health problem. The knowledge of bacterial communities in the gut of Vietnamese patients with T2DM and non diabetic controls is still insufficient. We report in this article the 16S rDNA amplicon data of the gut microbiomes of Vietnamese patients with T2DM and nondiabetic controls carried out using the Illumina sequencing. This work included 7 patients and 7 controls. A total of 1,627,646 reads were obtained and a total of 13 phyla, 25 classes, 94 genera were revealed. The top three dominant bacterial phyla in all subjects were Firmicutes, Bacteroidetes and Proteobacteria. Significant differences in the relative abundances of the phylum Firmicutes and class *Clostridia* between patients and controls were observed, suggesting that the reducing of phylum Firmicutes and class *Clostridia* in the gut may be linked to obesity and T2DM. All sequencing libraries were deposited in the NCBI SRA as BioProject PRJNA668251. The datasets are needed to determine the association between the bacterial composition of the gut and the pathogenesis of T2DM in Vietnamese patients.

## Specifications Table

**Table:** 

Subject	Microbiology
Specific subject area	Metagenomics
Type of data	Table Figure FASTQ files
How data were acquired	Amplicon metasequencing using the Illumina HiSeq platform
Data format	Raw and analysed
Parameters for data collection	Faecal samples were collected from adults over 24 years old. All participants provided written consent prior to investigation. All methods were performed in accordance with the Declaration of Helsinki. Type 2 diabetes patients (7 people), who have been diagnosed with type 2 diabetes for at least 5 years, were grouped with blood glucose, cholesterol, low-density lipoprotein cholesterol (LDL-C), high-density lipoprotein cholesterol (HDL-C), triglyceride, alanine amino transferase (AST) and aspartate amino transferase (ALT) levels that are outside the normal range and with body mass indices greater than 30. Nondiabetic adults (7 people) are those with above indices in the normal range and have body mass indices of less than 23.
Description of data collection	Total DNA was extracted from faecal samples, the V3-V4 variable regions of 16S rDNA were amplified, and paired-end sequenced according to the Illumina protocol using the Illumina platform.
Data source location Data accessibility	Institution: Institute of Biotechnology, Vietnam Academy of Science and Technology City: Hanoi Country: Vietnam Raw data were deposited to NCBI Repository name: SRA Data identification number: BioProject PRJNA668251, BioSamples from SAMN16425402 to SAMN16425415 Direct URL to data: https://www.ncbi.nlm.nih.gov/bioproject/?term=PRJNA668251

## Value of the Data

• This dataset provides the description of bacterial community in the gut of T2DM patients and non diabetic adults in Vietnam based on sequencing of 16S rDNA gene amplicons.
• Analysis of 16S rDNA sequences showed that the relative abundances of the phylum Firmicutes and class *Clostridia* were significantly lower in the T2DM patients than in the controls. This can help to verify the hypothesis that changing gut microbiota may contribute to Vietnamese T2DM.
• The data might be used for further bioinformatics processing and the datasets are useful for comparison with the gut microbiota of adults in other nations.

## 1 Data Description

**Fig. 1:**
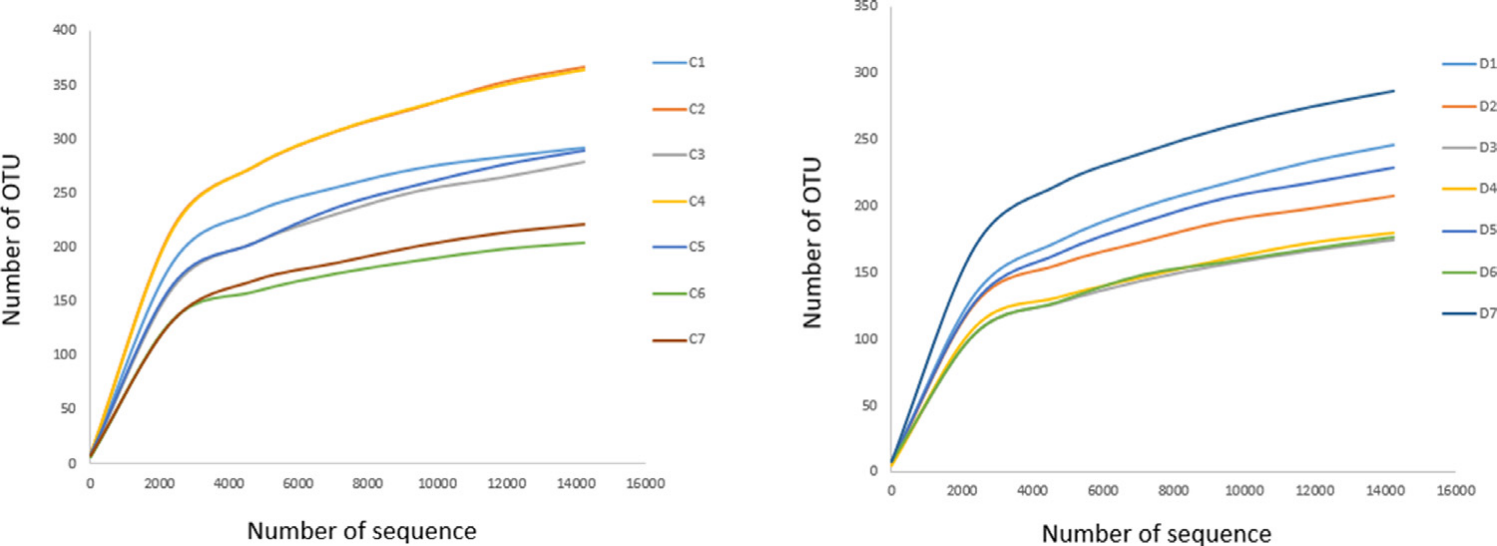
Rarefaction analysis of V3-V4 sequencing of the 16S rDNA gene in faecal samples from T2DM patients (D1–D7) and nondiabetic controls (C1–C7). Rarefaction curves were calculated from samples at 97% sequence identity of 16S rDNA gene.

To determine the bacterial population in the faeces of Vietnamese adults with T2DM (D1-D7) and nondiabetic controls (C1-C7), 16S rDNA gene amplicon sequencing was performed. A total of 1,627,646 reads were obtained from 14 subjects by sequencing the V3-V4 region of the 16S rDNA gene. The number of sequences varied between subjects, ranging from 34,411 to 134,397, with a mean of 116,260 (SD 25,494). The average length of qualified sequences was 417 bp. The total numbers of OTUs varied from 175 to 287 in the diabetic subjects and 204 to 367 in the controls. The mean bacterial diversities estimated by Chao1 indices in the diabetic subjects and the controls were 280 (SD 58) and 343 (SD 82), respectively (Table 1). However, the rarefaction curves did not approach the plateau for the two groups, indicating that bacterial richness has not yet been fully determined (Fig. 1).

**Table 1:** Summary of the sequences analysis and bacterial diversity indices: observed richness (OTUs), richness estimates (Chao1) within faecal samples of T2DM patients (D1-D7) and controls (C1–C7).

Sample ID	Age	Sequences produced	Sequences qualified	Average length (nt)	OTUs	Chao1
D1	70	128,305	103,614	423	246	363
D2	71	121,968	103,461	418	208	271
D3	66	102,246	66,980	416	175	243
D4	52	132,071	90,189	422	180	234
D5	55	119,757	100,316	420	229	292
D6	59	134,397	107,500	417	177	210
D7	69	123,799	104,777	418	287	352
C1	38	34,411	24,414	416	292	314
C2	31	120,516	102,192	417	367	465
C3	34	106,163	75,766	416	279	366
C4	43	127,451	109,208	415	364	418
C5	40	128,711	108,967	417	289	349
C6	39	134,182	96,465	416	204	237
C7	24	113,669	77,896	417	221	257

**Fig. 2:**
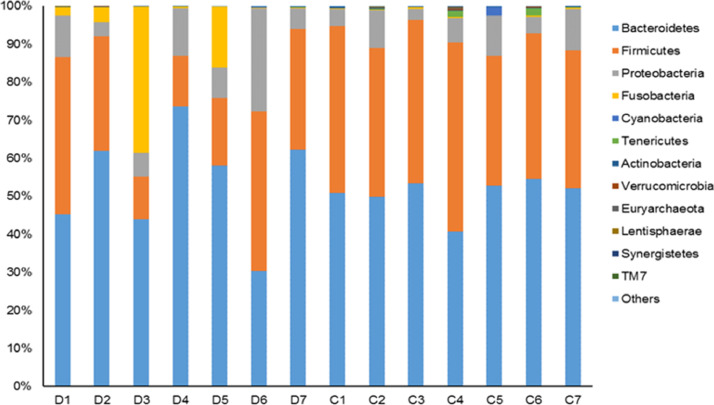
Relative abundances of bacterial phyla.

Bacterial reads in the two groups were classified into 13 major phyla, 25 classes, and 94 genera. Among them, Firmicutes and Bacteroidetes were the two most abundant phyla in all subjects, accounting for up to 96% of sequences, followed by Proteobacteria (Fig. 2). The relative abundance of Firmicutes was significantly lower (*P* = 0.021) in the diabetic group (mean 26.7 ± 12.75%) compared to the controls (mean 40.5 ± 5.23%). Within phylum Firmicutes, sequences belonged to class *Clostridia* were dominant, ranged from 11.18% to 48.33% between subjects. The percentage of class *Clostridia* was lower in the diabetic group compared to the healthy group (*P* = 0.009, mean of 25.5 ± 11.6% and 40.1 ± 4.94%, respectively).

**Fig. 3:**
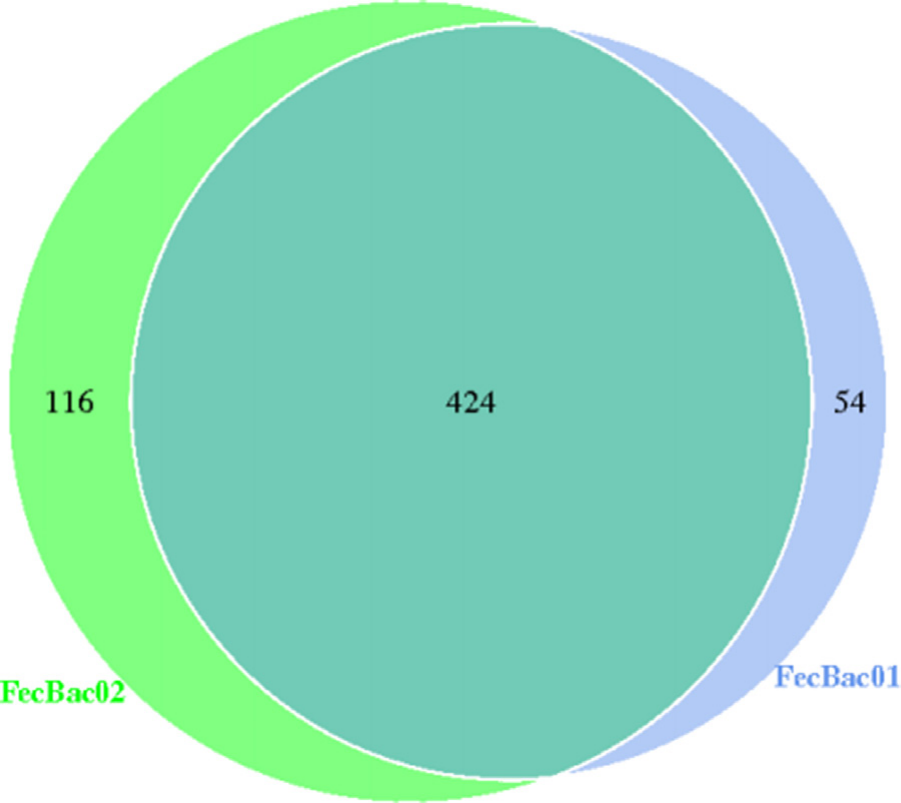
The shared OTUs in the gut between T2DM patients (FecBac01) and nondiabetic controls (FecBac02). The Venn diagram presents the unique and shared OTUs at the 97% similarity level in the guts of those with type 2 diabetes mellitus and nondiabetic controls.

Overlaps and differences in bacterial OTUs in the gut between T2DM patients and nondiabetic controls showed that adults with type 2 diabetes containing 478 OTUs and nondiabetic adults containing 540 OTUs shared 424 OTUs (Fig. 3).

Here, the first datasets on the bacterial diversity of the gut microbiome of adults with T2DM and nondiabetic adults from Vietnam were examined by 16S rDNA Illumina sequencing.

## 2 Experimental Design, Material and Methods

### 2.1 Experimental design

Patients selected to participate in this work are those with type 2 diabetes from 2012 and before (have been affected by type 2 diabetes for more than 5 years), with blood glucose, cholesterol, low-density lipoprotein cholesterol (LDL-C), high-density lipoprotein cholesterol (HDL-C), triglyceride, alanine amino transferase (AST) and aspartate amino transferase (ALT) levels outside the normal ranges and with body mass indices (body weight in kilograms, divided by the squared value in metres) greater than 30. Healthy people participating in this work had the above indices in the normal range and had body mass indices of less than 23.

Subjects with type 2 diabetes and nondiabetic controls were all females aged 24 to 70 years, with body mass indices (BMIs) ranging from 30.04 to 32.46 kg/m^2^ for those with type 2 diabetes and from 18.06 to 22.43 kg/m^2^ for the nondiabetic controls. The diabetic group had elevated concentrations of plasma glucose compared to the control group (8.90 ± 3.8 vs 5.24 ± 0.28).

Health status of two groups was evaluated by specialists from Transport Hospital Joint Stock Company. Median of white blood cell (WBC), red blood cell (RBC), and platelet counts were found to not significantly differ between T2DM and control subjects.

### 2.2 Sample collection

Faecal samples were collected in a sterile container and kept at +4 °C, were brought to the laboratory within 24 h, and were stored at −80 °C until processing. The faecal samples were obtained from 14 women with T2DM (D1-D7) and nondiabetic controls (C1-C7). Genomic DNA was extracted in triplicate (in each replicate 0.25 g of faecal sample was used) by a PowerFecal DNA Isolation Kit (catalogue No. 12,830–50, MO BIO, USA). The DNA concentration and quality were determined by agarose gel electrophoresis and using a NanoDrop spectrophotometer (ThermoFisher Scientific, Inc., Waltham, MA, USA).

### 2.3 Library preparation and sequencing

DNA mixtures containing equivalent amounts of DNA from the pooled samples were used for PCR. The V3-V4 regions of 16S rDNA gene were amplified using the specific primers 341F (5′-CCTAYGGGRBGCASCAG-3′) and 806R (5′-GGACTACNNGGGTATCTAAT-3′) [1], with specific six-nucleotide barcodes added at the 5′ end to allow multiple samples to be analysed in parallel. All PCR reactions were performed with Phusion® High-Fidelity PCR Master Mix (catalogue No. E0553L, New England Biolabs, UK). Sequencing libraries were generated by using TruSeq® DNA PCR-Free Library Preparation Kit (catalogue No. 20,015,962, Illumina, USA) according to the manufacturer's recommendations. The library quality was assessed by a Qubit@ 2.0 Fluorometer (Thermo Scientific, USA) and an Agilent Bioanalyser 2100 system (Agilent, USA). Then, paired-end 2 × 250-bp sequencing was carried out on an Illumina HiSeq 2500 platform (Illumina, USA).

### 2.4 Sequence data analysis

Based on the unique barcodes, paired-end reads were assigned to samples and were truncated by cutting off the barcode and primer sequences. Paired-end reads were merged using FLASH version 1.2.7 [2]. After assembly, quality filtering of the raw tags was performed using default filtering conditions according to the QIIME (Version 1.7.0) quality control process [3], [4], chimaeric sequences were detected and removed using the UCHIME algorithm. Sequence processing was performed using UPARSE software v7.0.1001 [5]. OTUs were determined using a similarity threshold level of 97% between sequences. A representative sequence for each OTU was used for species annotation with the GreenGenes Database [6] based on the RDP (Version 2.2) classifier algorithm [7]. Subsequent analyses of alpha diversity and beta diversity were all performed based on the rarefied OTU table. Diversity indices were calculated with QIIME (Version 1.7.0) and displayed using R software (Version 2.15.3). To estimate and compare bacterial diversity in humans with T2DM and nondiabetic controls, OTUs from each sample were used to calculate three diversity indices: observed richness (OTUs), and Chao1 were calculated with the distance matrixes of weighted and unweighted UniFrac distances.

All sequencing libraries generated in this article were deposited in the NCBI GenBank database under the accession numbers SAMN16425402 to SAMN16425415.

## CRediT Author Statement

Hung The Hoang: Investigation, Validation, Writing - Original Draft, Writing - Review & Editing. Duc Hoang Le: Methodology, Investigation, Resources, Software, Formal analysis, Data Curation, Writing - Original Draft, Writing - Review & Editing. Thi Thanh Huyen Le: Sampling, Investigation, Resources, Writing - Original Draft. Thi Tuyet Nhung Nguyen: Sampling, Investigation, Resources, Writing - Review & Editing. Ha Hoang Chu: Supervision, Resources, Writing - Review & Editing. Nam Trung Nguyen: Supervision, Project administration, Funding acquisition, Writing - Original Draft; Writing - Review & Editing.

## Ethics Statement

Informed consent of the participants has been obtained prior to investigation. The sampling was carried out in accordance with the recommendations of the Ethics Committee of Institute of Biotechnology and performed according to the declaration of Helsinki.

## Funding

This work was funded by project NV03-PTNTĐ2017 (for Nam Trung Nguyen) from National Key Laboratory of Gene Technology, Institute of Biotechnology, Vietnam Academy of Science and Technology.

## Declaration of Competing Interest

The authors declare that they have no known competing financial interests or personal relationships which have or could be perceived to have influenced the work reported in this article.

## Appendix Supplementary materials

Image, application 1
