# Supplementary material for: Metagenomic 16S rDNA amplicon data of microbial diversity of guts in Vietnamese humans with type 2 diabetes and nondiabetic adults
Source: Data Brief. 2020 Dec 24;34:106690. doi: 10.1016/j.dib.2020.106690 (PMC7776958; doi:10.1016/j.dib.2020.106690)
Supplement: Supplementary file 1 [file mmc1.pdf]

Researcher Academy researcheracademy.com

ELSEVIER

Certificate of Completion

This certifies that

Hung Hoang

has successfully completed the following module

How do editors look at your paper?

on Thursday 08 October, 2020

Presented by Jaap van Harten

Suzanne BeDell
Managing Director, Education
Reference & Continuity Books

Philippe Terheggen
Managing Director, Science,
Technology & Medical Journals
